# DNA prime and peptide boost immunization protocol encoding the *Toxoplasma gondii* GRA4 induces strong protective immunity in BALB/c mice

**DOI:** 10.1186/1471-2334-13-494

**Published:** 2013-10-23

**Authors:** Min Meng, Aihua Zhou, Gang Lu, Lin Wang, Guanghui Zhao, Yali Han, Huaiyu Zhou, Hua Cong, Qunli Zhao, Xing-Quan Zhu, Shenyi He

**Affiliations:** 1 Department of Parasitology, Shandong University School of Medicine, Jinan, Shandong Province, 250012, Peoples Republic of China; 2 Department of Pediatrics, Provincial Hospital Affiliated to Shandong University, Shandong University School of Medicine, Jinan, Shandong Province, 250021, Peoples Republic of China; 3 State Key Laboratory of Veterinary Etiological Biology, Key Laboratory of Veterinary Parasitology of Gansu Province, Lanzhou Veterinary Research Institute, Chinese Academy of Agricultural Sciences, Lanzhou, Gansu Province, 730046, Peoples Republic of China

## Abstract

**Background:**

*Toxoplasma gondii* is a widespread intracellular parasite, which infects most vertebrate animal hosts and causes zoonotic infection in humans. Vaccine strategy remains a promising method for the prevention and control of toxoplasmosis. *T. gondii* GRA4 protein has been identified as a potential candidate for vaccine development. In our study, we evaluated the immune response induced by four different immunization vaccination strategies encoding TgGRA4.

**Methods:**

BALB/c mice were intramuscularly (i.m.) immunized four times according to specific immunization schedules. Generally, mice in experimental groups were immunized with polypeptide, pGRA4, peptide/DNA, or DNA/peptide, and mice in the control groups were injected with PBS or pEGFP. After immunization, the levels of IgG antibodies and cytokine productions were determined by enzyme-linked immunosorbent assays (ELISA). The survival time of mice was also evaluated after challenge infection with the highly virulent *T. gondii* RH strain.

**Results:**

The results showed that mice vaccinated with different immunization regimens (polypeptide, pGRA4, peptide/DNA, or DNA/peptide) elicited specific humoral and cellular responses, with high levels of total IgG, IgG2a isotype and gamma interferon (IFN-γ), which suggested a specific Th1 immunity was activated. After lethal challenge, an increased survival time was observed in immunized mice (11.8 ± 4.8 days) compared to the control groups injected with PBS or pEGFP (P < 0.05). Mice injected with PBS or pEGFP died within 8 days, and there was no significant difference in the protection level in two groups (P > 0.05).

**Conclusions:**

These results demonstrated that this DNA prime and peptide boost immunization protocol encoding the TgGRA4 can elicit the highest level of humoral and cellular immune responses compared to other immunized groups, which is a promising approach to increase the efficacy of DNA immunization.

## Background

*Toxoplasma gondii* is a widespread intracellular parasite belonging to the phylum Apicomplexa, which infects most vertebrate animal hosts and causes zoonotic infection in humans [1]. In general, an asymptomatic but chronic infection is established in immunocompetent individuals; However, in selected immunocompromised human, and in particular those with human immunodeficiency virus (HIV) infection, *T. gondii* can result in the extensive and fatal tissue damage [2]. The life cycle of *T. gondii* consists of two phases: the sexual stage only in felines and the asexual stage in human and other intermediate hosts [3]. The asexual component comprises two distinct stages of growth: rapidly growing 'tachyzoites’ and latent 'bradyzoite’ tissue cysts [4]. Host cell invasion and lysis by the actively dividing tachyzoites are directly responsible for toxoplasmosis, which is particularly severe in immunocompromised individuals and in the congenitally infected fetus [5,6].

Although drugs are the primary strategy for the treatment of toxoplasmosis, they are poorly tolerated, have severe side effects and drug-resistance, and cannot act against chronic *T. gondii* infection [3]. It is known that individual patients infected with *T. gondii* have serious consequences and the disease burden of congenital toxoplasmosis on a population level is considerably high [7]. Thus, the development of an effective and safe vaccine against *T. gondii* acute and chronic infection is an important and urgent goal. So far, only a commercial vaccine is deployed to control toxoplasma abortion in sheep, which comprises live tachyzoites of the S48 'incomplete’ strain of *T. gondii*[8]. In human, an effective vaccine could be valuable for preventing both fetal infection and reactivation in immunocompromised individuals; In livestock such as sheep and goats, it could prevent spontaneous abortion, decrease economic losses and reduce the potential for a major epidemiologic vector for human infection [9,10]. In general, proteins or peptides and DNA vaccines are considered as the third generation subunit vaccines, which are important in preventing intracellar parasites infection [11]. DNA vaccines encoding specific antigen can activate all pathways of immune response, with production of antibody, Th1-dominated CD4+ T cells and CTLs (cytotoxic T-cell responses), and provide protection against diseases that require cell-mediated immunity such as intracellular protozoan infections [12]. DNA vaccines against *T. gondii* have been proved that they can induce antibody, specific T-cell responses and protective immunity against acute and chronic challenge in mice [13,14]. In contrast to DNA vaccines, synthetic multiple antigen peptide (MAP) vaccines are an effective and new approach to deliver multiple T-cell and B-cell epitopes as the constituents of a single immunogen, which contains a high concentration of the relevant antigen for inducing immune responses to predefined epitopes [15]. Synthetic MAP vaccines have been shown to elicit better cell-mediated immunity by focusing the host immune response on epitopes known to play a role in protective immunity [16,17]. Recently, several studies have demonstrated the power of synthetic polypeptides vaccines in eliciting protective immunity to intracellular parasites such as *Plasmodium falciparum*[18], *Schistosoma mansoni*[19], *T. gondii*[17], and *Paracoccidioides brasiliensis*[20].

Lots of studies indicate that the family of promising vaccine candidate antigens includes surface antigen (SAGs) [21], dense granule proteins (GRAs) [22], rhoptry proteins (ROPs) [23], and micronemal proteins (MICs) [24]. GRA proteins, highly expressed by *T. gondii*, are involved in parasite survival and virulence and are the major member of the excreted secreted antigens (ESA) [25,26]. Among the GRA proteins, GRA4 has been identified as the leading candidate for vaccine development [27]. In mice immunized with plasmids encoding for GRA4 antigen or the recombinant GRA4 protein generated a Th1 type immune response and provided partial protection against parasite infection [28,29]. Concerning GRA4 peptides, amino acids 229-242 and 231-245 containing B and T-cell epitopes can induce both humoral and cellular immune responses following oral infection with *T. gondii*[30,31]. Multi-epitope DNA vaccine expressing six antigen segments of SAG1, GRA1 and GRA4 produced stronger humoral and Th1-type cellular immune responses, which is a potential strategy for the control of toxoplasmosis [32]. A multiple antigenic peptide (MAP) vaccine including one B-cell and two T-cell epitopes derived from *T. gondii* antigens (SAG1, GRA4 and GRA1) could trigger stronger humoral and cellular responses against *T. gondii*[17]. Therefore, GRA4 is an attractive and promising vaccine candidate against *T. gondii* and GRA4 231-245 peptide containing B and T-cell determinants has been proved to be immunogenic, and was considered suitable alternative in polypeptide vaccine design.

Furthermore, vaccination strategy is also a key factor in influencing immunity, which is as important as vaccine candidates [33]. One particularly promising approach is the prime-boost strategy, which has been shown to generate high level of T-cell memory in animal models [34]. To increase antibody production following DNA immunizations, prime-boost regimens have been shown to be an effective approach to induce both humoral and cellular immune responses [35,36]. The key strength of this strategy is that greater levels of immunity are established by heterologous prime-boost in which the antigen is applied via different routes and immunization sites [34].

The aim of the present study is to investigate their protective efficacy of different immunization regimens (polypeptide, pGRA4, peptide/DNA, or DNA/peptide) in mice against lethal *T. gondii* challenge. Our results demonstrate that the DNA prime-peptide boost vaccination regime can elicit the highest level of humoral and cellular immune responses, and is an effective strategy in increasing efficacy of DNA immunization.

## Methods

### Mice and parasite

Specific-pathogen-free (SPF) grade female Kunming and BALB/c mice (6-8 weeks old) were purchased from Shandong University Laboratory Animal Centre (Jinan, China). In this study, the *T. gondii* RH strain (Type I), a highly virulent strain for mice, was used to challenge immunized BALB/c mice. Tachyzoites of the RH strain was maintained in liquid nitrogen and recovered routinely by intraperitoneal passage in Kunming mice in our laboratory according to the method previously described [37]. Tachyzoites of *T. gondii* were harvested from the peritoneal fluid of Kunming mice that had been intraperitoneally infected three days earlier. Soluble tachyzoite antigen (STAg) was obtained from RH strain tachyzoites as previously described [38]. Briefly, parasites obtained from the peritoneal exudates were washed three times by centrifugation, then suspended in sterile phosphate buffered saline (PBS) and sonicated for three 10 min periods at 60 w/s. The toxoplasma sonicate was centrifuged at 2100 × g for 15 min. The supernatant, containing soluble tachyzoite antigens (STAg), was harvested and stored at -80°C until further use.

### Construction of the eukaryotic expression plasmid

The total genomic DNA of *T. gondii* RH strain was extracted from purified tachyzoites using a commercial kit (QIAGEN, Germany) and used as a template DNA for the polymerase chain reaction (PCR). The coding sequence of the *T. gondii* GRA4 gene [GenBank: AAA30142.1] was amplified from the *T. gondii* genomic DNA, with a pair of specific synthetic primers (forward primer: 5′- CGGGGTACCATGCAGGGCACTTGGTTTTC -3′, reverse primer: 5′- CGCGGGATCCTCACTCTTTGCGCATTCTTT -3′), in which KpnI and BamHI restriction sites were introduced. The PCR conditions were as follows: pre-denaturation at 94°C for 5 min; denaturation at 94°C for 30 s, annealing at 65°C for 30 s and extension at 72°C for 1 min, followed by 30 cycles; final extension at 72°C for 10 min. The sequence of amplified GRA4 DNA fragment was verified by double-stranded sequencing. The GRA4 DNA fragment was subcloned into the eukaryotic expression vector pEGFP-C1 (Clontech, USA) to form the plasmid pEGFP-GRA4 using KpnI and BamHI restriction sites. The recombinant plasmids were then transformed into *Escherichia coli* DH5α. Positive recombinant plasmids were verified using PCR, double restriction enzyme digestion and double-stranded sequencing. The plasmids were purified using an endotoxin-free plasmid purification kit following the manufacturer’s instructions, and stored at -20°C until use. The concentration of pGRA4 was determined by spectrophotometer at OD 260 and OD 280, and the ratios were 1.8-2.0.

### Design and synthesis of peptide

Peptide constructs were synthesized, purified, and identified as described previously [17,39]. Briefly, MAP vaccine of GRA4 was designed based on the published peptide sequences containing T-cell epitopes and B-cell epitopes [17,40]. In this study, peptide 231-245 (STEDSGLTGVKDSSS) was selected and polypeptides were synthesized by Xi’an Bio-scientific Co., Ltd with the Solid-Phase Peptide Synthesizer by adopting four-branched peptide design. The purity of the peptide was confirmed by analytic HPLC.

### pEGFP-GRA4 plasmid expression *in vitro*

HEK293T cells were maintained in our laboratory and routinely cultured in Dulbecco’s modified Eagle’s medium (DMEM) supplemented with penicillin (100 IU/ml), streptomycin (100 mg/ml) and 10% fetal bovine serum (FBS) at 37°C with 5% CO_2_. The *in vitro* transfection assay was performed as previously described [41]. Briefly, before transfection, 2 × 10^4^ HEK293T cells were seeded in a 6-well plate. The recombinant pGRA4 plasmid (2 μg/well) was transfected into cells using the Fugene HD transfection reagent as instructed by the manufacturer. pEGFP plasmid-transfected cells and untransfected cells were used as the controls. Plates were incubated for 48 h at 37°C in 5% CO_2_, 95% humidity. The plasmid pGRA4 expression in the cells was detected by western blotting analysis.

Protein productions from HEK293T cells were collected on ice with RIPA Lyses Buffer (50 mM Tris pH 7.4, 150 mM NaCl, 1% Triton X-100, 1% Sodium deoxycholate, 0.1% SDS) containing 1 mM protease inhibitor PMSF (phenylmethanesulfonyl fluoride). The expression of plasmid pGRA4 in HEK cells were demonstrated by western blotting with anti-*T. gondii* polyclonal antibody (Goat) and a HRP (horseradish peroxides)-labeled rabbit anti-goat IgG antibody (Sigma, USA) as a secondary antibody. The conjugated substrate was visualized with ECL chemiluminescence’s reagents as described previously [13], and western blot marker (CWBIO, China) was used as molecular mass standards.

### Immunization and challenge

Six groups of BALB/c mice (n = 12 each) were individually immunized intramuscularly (i.m.) four times at two weeks interval. The specific immunization schedules and methods for mice are shown in Table 1. Blood samples from each group were collected from the tail vein at the end of each interval, four times in total and sera were separated and stored at -20°C until analyzed for specific antibodies. Two weeks after the last immunization, three mice per group were sacrificed and splenocytes were harvested under aseptic conditions for cytokine assays, and thereafter the remaining mice in all groups were intraperitoneally (i.p.) challenged with 1 × 10^3^ *T. gondii* RH strain tachyzoites suspended in 100 μl phosphate-buffered saline (PBS) to observe the percentage of mice surviving.

**Table 1:** Specific immunization regimes of BALB/c mice

**Groups**^**a**^	**Immunization time**			
	**1**^**st**^	**2**^**nd**^	**3**^**rd**^	**4**^**th**^
PBS	100 μl	100 μl	100 μl	100 μl
pEGFP^b^	100 μg	100 μg	100 μg	100 μg
pGRA4^c^	100 μg	100 μg	100 μg	100 μg
peptide^d^	100 μg	100 μg	100 μg	100 μg
peptide/DNA	100 μg peptide	100 μg peptide	100 μg pGRA4	100 μg pGRA4
DNA/peptide	100 μg pGRA4	100 μg pGRA4	100 μg peptide	100 μg peptide

^a^All mice of six groups were intramuscularly injected four times at two week interval.

^b^Each mouse, 100 μg pEGFP resuspended in 100 μl PBS.

^c^Each mouse, 100 μg pGRA4 resuspended in 100 μl PBS.

^d^Each mouse, 100 μg peptide resuspended in 100 μl PBS.

### Determination of antibodies by ELISA

IgG, IgG1 and IgG2a antibodies in sera samples were determined using an indirect ELISA assay according to the manufacture’s instructions (R&D Systems, USA). In brief, the 96-well microtiter plates were coated over night at 4°C with STAg in 50 mM carbonate buffer (pH 9.6). On the second day, the plates were washed three times with PBS containing 0.05% Tween20 (PBST) and then blocked with 1% Bovine Serum Albumin (BSA) for 1 h at 37°C. Thereafter, the wells were washed and incubated with mice sera diluted in PBS for 1 h at 37°C. HRP-conjugated goat anti-mouse IgG, IgG1 or IgG2a diluted in PBS (Sigma, USA) were used as the secondary antibody and added to the wells for determination of antibody levels and isotype analysis, respectively. Finally, immune complexes were visualized by incubating with orthophenylene diamine (Sigma, USA) and 0.15% H_2_O_2_ for 30 min. The reaction was stopped by adding 2 M H_2_SO_4_, and the absorbance was measured at 490 nm with an ELISA reader (BiotekELx800, USA). All samples were run in triplicate.

### Cytokine assay

To assay for the levels of cytokine productions, spleens were aseptically removed from three mice per group two weeks after the last injection and splenocytes were obtained as described above and cultured in 96-well microtiter plates. Cell-free supernatants were harvested from microtiter plates and assayed for cytokine levels. The assays were performed for interleukin-4 (IL-4) activity at 24 h, interleukin-10 (IL-10) activity at 72 h, and gamma interferon (IFN-γ) activity at 96 h using a commercial ELISA kit ( R&D Systems, USA) following the manufacturer’s recommendations. All samples were performed in triplicate.

### Statistical analysis

Statistical analysis and graphics were performed using SPSS software. All data, including antibody levels and cytokine productions, were compared between the different groups by one-way ANOVA. Survival time among all the groups was compared using the Kaplan-Meier method. The results in comparisons were considered different if P < 0.05.

### Ethics statement

This study was approved by the Institutional Animal Care and Use Committee of Shandong University under Contract 2011-0015, and the animals were kept and the experiments were performed in accordance with committee’s criteria for the care and use of laboratory animals. All mice were maintained in specific pathogen-free conditions, and all efforts were made to minimize suffering. Humane endpoints to reduce pain or distress in mice were used via euthanasia. Mice were monitored daily for signs of toxoplasmosis, which included difficulties with their food and water intake, lethargy, or severe ascites. Mice with above signs were sacrificed immediately using CO_2_ gas. Generally, mice were placed in a chamber and CO_2_ was administered at a concentration of 60% to 70% over a 5-minute exposure time, after which the cervical dislocation method was sometimes used to ensure that effective euthanasia had occurred.

## Results

### Expression of plasmid pGRA4 in HEK293T cell

**Figure 1:**
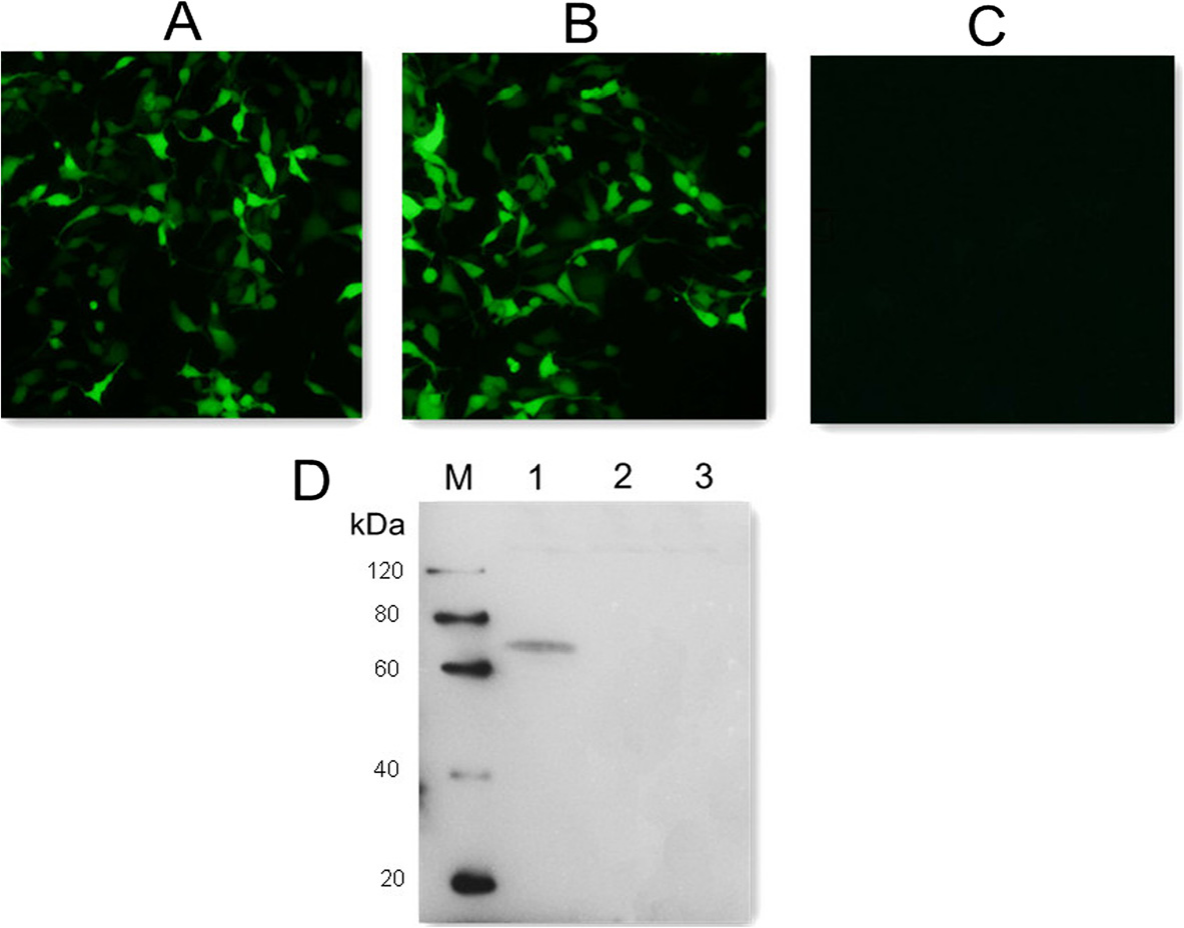
**Fluorescence microscopy images of HEK293T cells and western blotting analysis. (A)** HEK293 cells transfected with empty vector pEGFP-C1. **(B)** HEK293 cells transfected with pGRA4. **(C)** untransfected HEK cells. **(D)** protein marker (lane M), pGRA4-transfected cells (lane 1), pEGFP-transfected cells (lane 2), untransfected cells (lane 3).

Forty-eight hours after HEK cells were transfected with pGRA4 or empty plasmid pEGFP, specific green fluorescence was observed under a fluorescence microscope, whereas there was no signal in the untransfected cells (Figure 1A, B & C). In the western blotting analysis (Figure 1D), a specific protein band (about 70 kDa) was detected in cells transfected with pGRA4, but not in cells transfected with empty plasmid or untransfected cells. The fusion protein of about 70 kDa consists of the target protein GRA4 (about 40 kDa) and green fluorescent protein (about 30 kDa). These results indicated that the recombinant plasmid was successfully constructed and expressed *in vitro* and recombinant TgGRA4 protein possessed immunological activity.

### Evaluation of the antibody responses by ELISA

**Figure 2:**
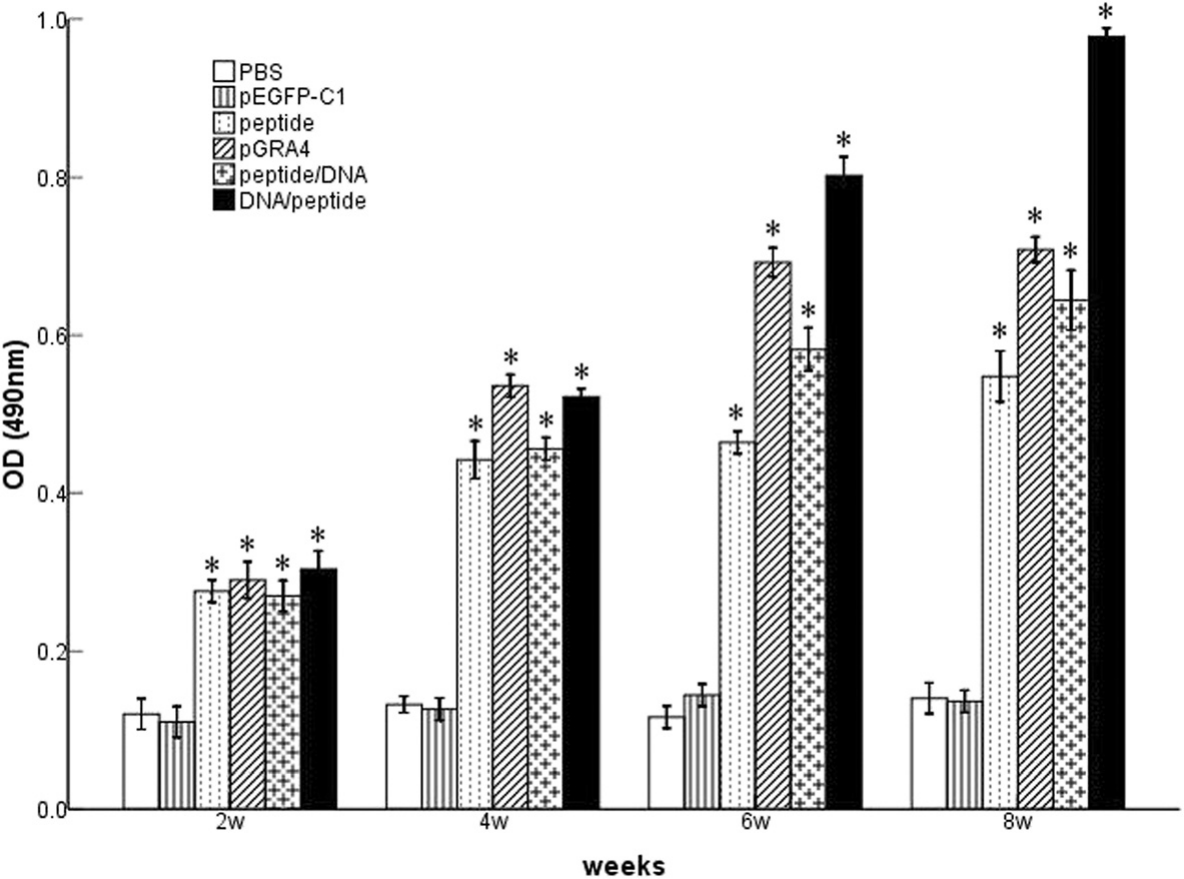
**Levels of specific IgG antibodies in BALB/c mice.** Sera samples of mice were collected from the tail vein at the end of each interval, four times in total and analyzed by ELISA. Results are shown as mean ± SD and statistically significant differences (P < 0.05) are indicated by (*) as compared to control groups.

To determine the specific antibody titers in all vaccination protocols, the levels of *T. gondii*-specific IgG and IgG subclasses in the sera of mice were assayed by ELISA with STAg as coating antigens. As shown in Figure 2, significantly high levels of IgG antibody appeared following injection of polypeptide, pGRA4, peptide/DNA, and DNA/peptide, as compared to the control groups (P < 0.05). As expected, mice injected with PBS and pEGFP did not generate specific antibody response. In all vaccination protocols, the DNA prime-peptide boost regimen leads to remarkable increase in specific IgG antibody production compared to other immunized groups (polypeptide, pGRA4, or peptide/DNA) (P < 0.05). However, there was no statistical difference in mice vaccinated with polypeptide, pGRA4, or peptide/DNA (P > 0.05).

**Figure 3:**
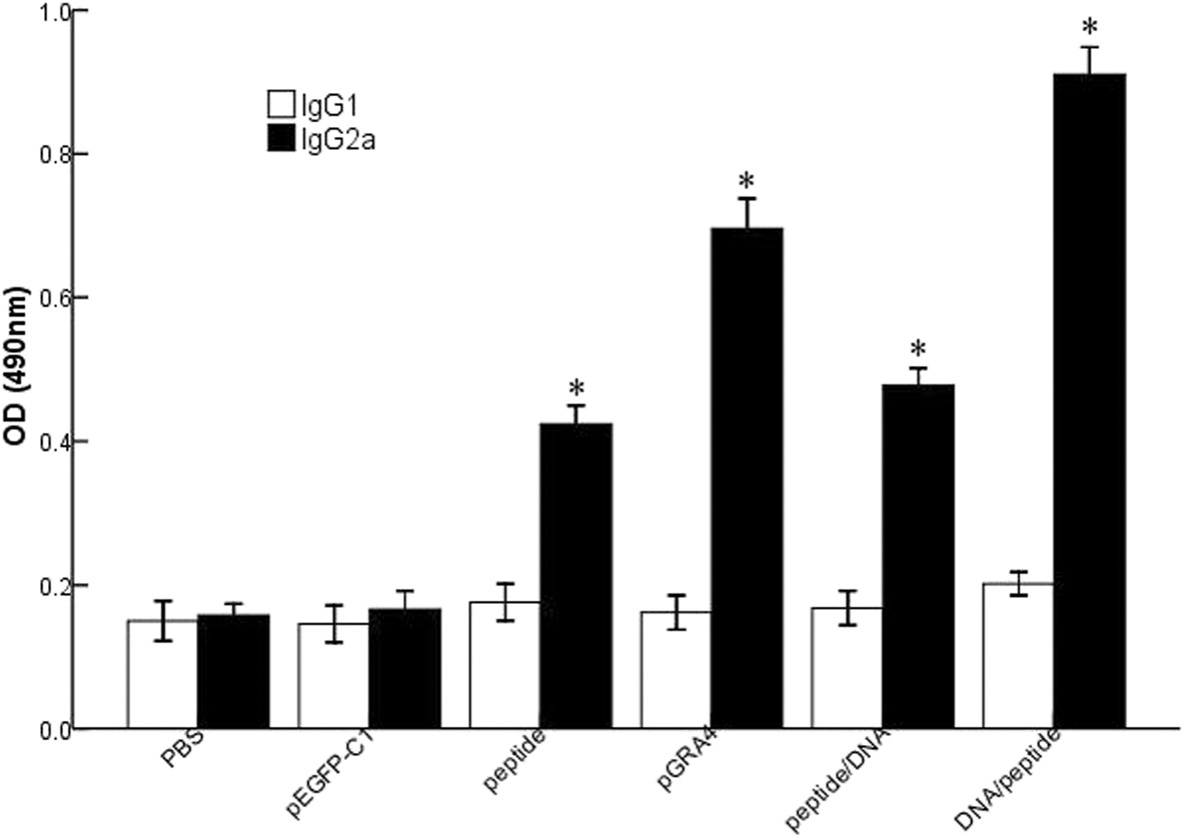
**Determination of IgG subclass IgG1 and IgG2a in the sera of BALB/c mice.** Sera were taken from mice in all groups two weeks after the last immunization and was analyzed by ELISA. Statistically significant differences (P < 0.05) are indicated by an asterisk (*) as compared to control groups.

In order to determine whether a Th1 and/or Th2 response was elicited in the immunized mice, we measured by ELISA the specific IgG1 and IgG2a subclasses at the second week after the final immunization. As depicted in Figure 3, an IgG2a predominant production over IgG1 was detected in the sera of vaccinated mice. Apparently, in mice immunized with DNA/peptide, the level of IgG2a was significantly increased (P < 0.05), compared with mice immunized with peptide/DNA (P < 0.05). This profile of IgG2a over IgG1 suggested that the heterologous DNA prime-peptide boost strategy elicited a specific humoral response, and with a Th1-type immune response.

### Cytokine production by spleen cells

**Figure 4:**
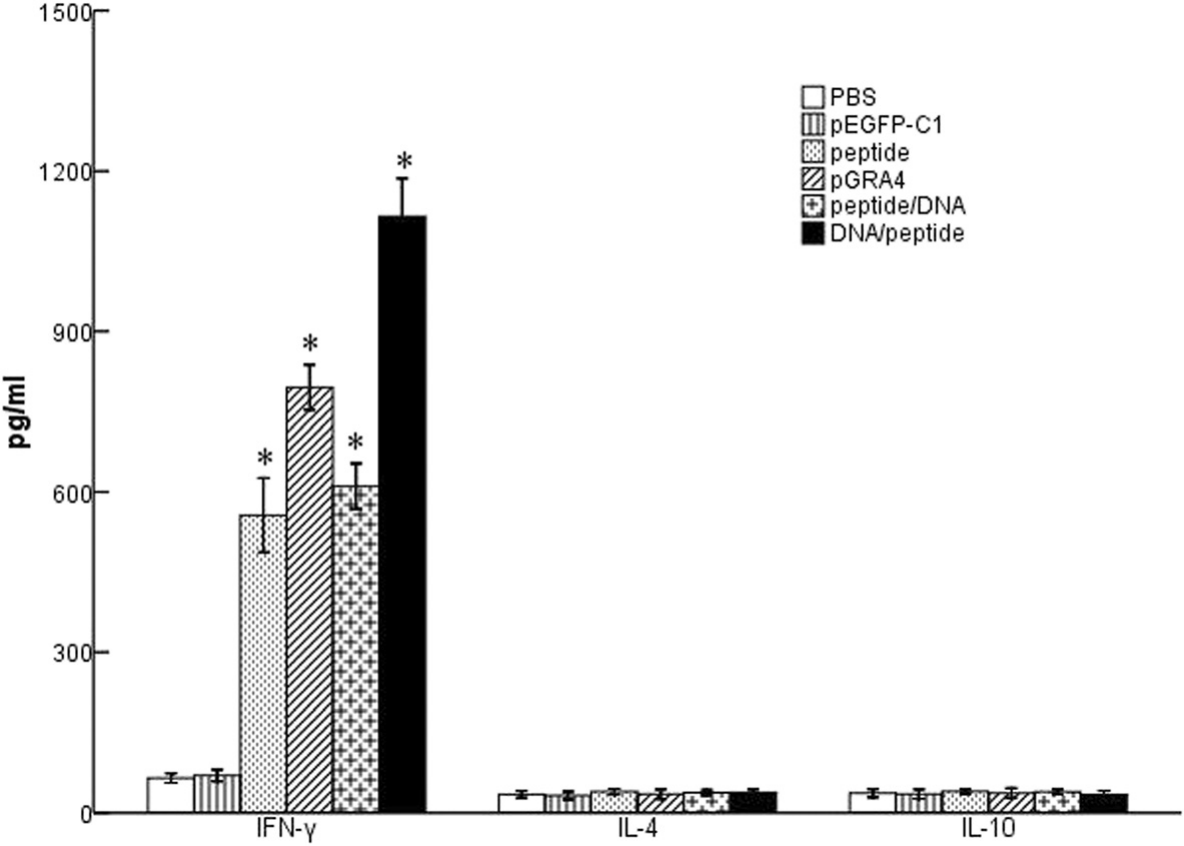
**Cytokine productions by spleenocyte cultures from mice.** Splenocytes were collected from three mice per group two weeks after the final immunization to detect the level of IFN-γ, IL-4 and IL-10. Data are expressed as mean ± SD. Statistically significant differences (P < 0.05) are indicated by (*) compared with negative control.

Culture supernatants of splenocytes from individual mice were harvested two weeks after the final vaccination and were assessed for the production of IFN-γ, IL-4 and IL-10 activities at different times. As shown in Figure 4, large amounts of IFN-γ were detected in the restimulated splenocyte cultures of all the treatment mice as compared to the control groups (P < 0.05). Mice vaccinated with DNA/peptide generated higher level of IFN-γ compared with pGRA4 or polypeptide alone (P < 0.05). In the meanwhile, no significant difference was observed in IL-4 and IL-10 levels between these groups (P > 0.05). Generally, both Th1 cytokines (IFN-γ and IL-2) and Th2 cytokines (IL-4 and IL-10) are the major parameters to define whether a Th1 and/or Th2 immune response was induced [22]. The high level of IFN-γ and low level of IL-4 and IL-10 suggested that the cellular immune response induced by the heterologous DNA prime-peptide boost strategy was an enhanced, Th1-biased immunity, further confirming the results of the IgG subclass as shown above.

### Evaluation of the survival time in BALB/c mice

**Figure 5:**
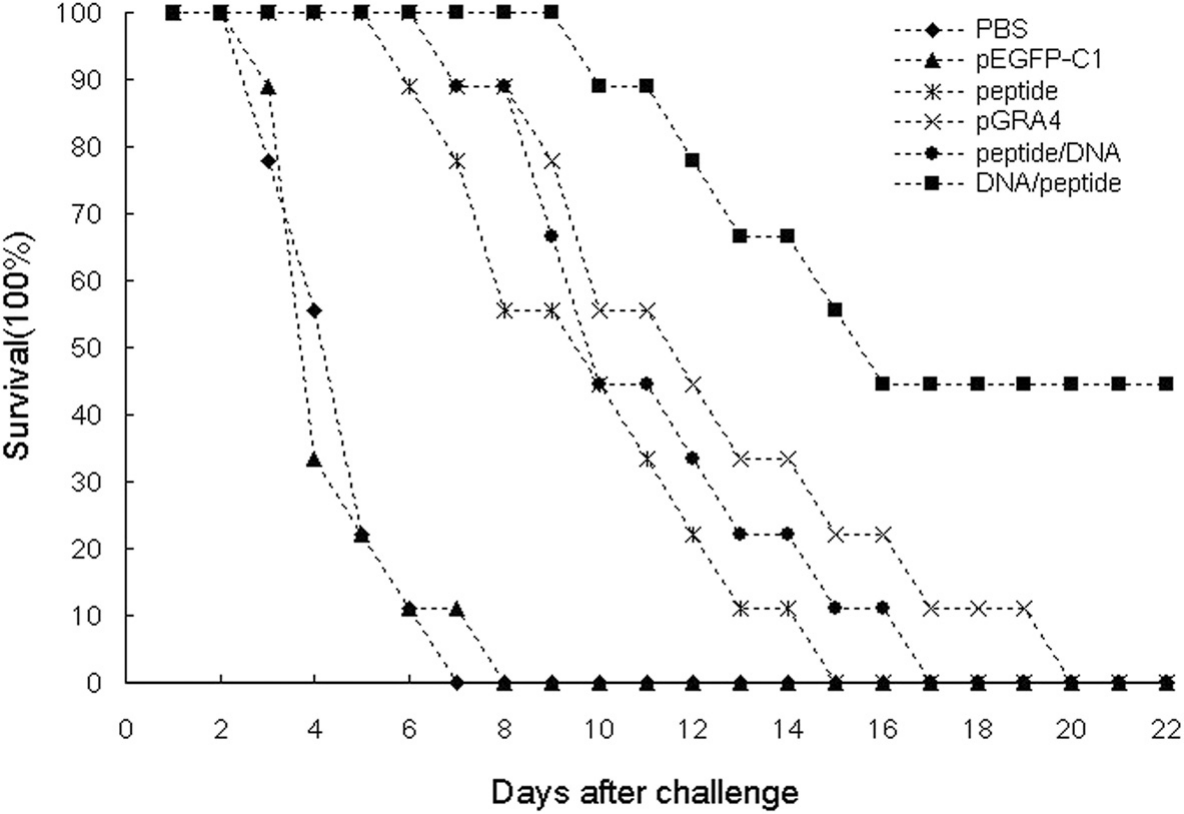
**Survival curves of BALB/c mice in all groups after *T. gondii *acute challenge.** Each mouse was challenged intraperitoneally with 1 × 10^3^ *T. gondii* RH strain tachyzoites at two weeks after the last immunization. Survival times were monitored daily after the challenge. Each group was composed of nine mice.

To evaluate the protective effect of different vaccination protocols against *T. gondii* infection, mice in all groups were intraperitoneally challenged with 1 × 10^3^ tachyzoites of the virulent RH strain two weeks after last immunization. Survival curves of those different groups of mice are shown in Figure 5. An increased survival time was observed in immunized mice (11.8 ± 4.8 days) compared to the control groups injected with PBS or pEGFP (P < 0.05). Mice injected with PBS or pEGFP died within 8 days, and there was no significant difference in the protection level in two groups (P > 0.05). Mice immunized with DNA /peptide provide significantly higher protection, with remarkable increase in survival time (16.5 ± 5.4 days), and 40% survival rate was achieved after a lethal challenge, as compare with pGRA4 (11.5 ± 4.1 days), polypeptide (9.0 ± 3.0 days), or peptide /DNA (10.3 ± 3.2 days) (P < 0.05). These results demonstrated DNA prime-peptide boost protocol is an effective approach to produce protection against *T. gondii* acute infection.

## Discussion

In the present study, we have compared the protective efficacy of different immunization strategies in BALB/c mice. Our results suggested that all the vaccination regimens can trigger a significantly higher level of humoral and cellular immune responses than the control groups injected with PBS or pEGFP. Especially, the regime with DNA vaccine priming followed by peptide boosting elicited significantly high levels of IgG, IgG2a and IFN-γ secretion, which is an effective approach to provide protection against lethal *T. gondii* infection.

Immunotherapy may be one of the most promising approaches to prevent the infection of *T. gondii*. DNA vaccines encoding TgGRA4 often induce a specific Th1-polarized immune response, but DNA vaccine is often poorly immunogenic, and cannot produce enough protection against *T. gondii* challenge [28,42]. Heterologous prime-boost strategies have emerged as a powerful vaccination approach for inducing very strong cellular immunity, which involve the administration of two different vaccines expressing the same antigen, given several weeks apart [43]. The mechanism of prime-boost regimen is that the first immunization initiates the priming of the immune response and subsequent immunizations trigger further expansion of antigen specific cells and selection of cells with high antigen avidity to boost the specific responses [11]. Recently, several reports have demonstrated the efficacy of heterologous prime-boost vaccination strategies in generating protective immune response to a variety of pathogens, including malaria [44], *Leishmania*[45], and hepatitis C virus [46]. To overcome a significantly lower antibody titer following DNA immunization, prime-boost strategies have been used to increase immune responses to a number of DNA vaccines [47]. Concerning GRA4, DNA prime and a vaccinia virus boost immunization regimen has been evidenced and can induce effective immune responses [35]. Priming with DNA and boosting with polypeptide is another new and promising approach. In our present study, DNA prime-peptide boost immunization regimen encoding TgGRA4 has been proved to be very effective for eliciting humoral and cellular responses in BALB/c mice.

Humoral and cellular immunity play an important role in host resistance against *T. gondii.* Primary infection with *T. gondii* results in a strong and persistent Th1-type immune response, defined by the high level of IgG antibody response and large amounts of cytokines, which are essential for the control of infection [48,49]. Their findings have suggested that a good immunization protocol should be able to direct the T-helper cells toward a Th1 rather than a Th2-type response [50]. Regard to the roles of humoral immunity, specific IgG antibodies play a protective role in controlling *T. gondii* chronic infection in collaboration with macrophages and preventing reactivation because IgG antibodies can inhibit the attachment of the parasite to the host cell receptors [51]. In this study, only the immunized mice had remarkable levels of serum antigen-specific IgG antibodies, with a high ratio of IgG2a to IgG1 antibody titers. A significant higher level of IgG antibodies responses was detected in mice immunized with DNA/peptide as compared with other groups immunized with peptide, pGRA4 or peptide/DNA (P < 0.05). These results indicated that specific Th1 cells were mainly activated, which was further confirmed by analysis of the cytokine productions.

To further characterize the polarization of the immune response, the levels of cytokine productions (IFN-γ, IL-4 and IL-10) from spleen cells of mice were evaluated. Generally, cytokines play an important role in host resistance against *T. gondii*. IFN-γ-dependent cell-mediated immunity was important in early infection and control the replication of the protozoan [52]. Meanwhile, IFN-γ is of primary importance in restricting the growth of *T. gondii* in the acute phase of the infection and preventing reactivation of parasites from dormant cysts [53]. Furthermore, a previous study suggested that IFN-γ correlates with the differentiation of Th1 cells and IL-4 induces the development of Th2 cells [54]. Therefore, IFN-γ can be considered as a marker for protective immunity against *T. gondii*. Meanwhile, studies suggested the level of Th2-associated cytokines (IL-4 and IL-10) produced in *T.* go*ndii* infection play an important role in immune response. IL-4 appear relatively late after infection, which perhaps prevents the host from succumbing to early Th1-polarized hyperactive immune response that can be detrimental to the host, and high level of IL-4 was known to antagonize the production of IFN-γ [55,56]. Under our experimental conditions, high level of IFN-γ production was induced in the experimental mice as compared to the controls, whereas the productions of IL-4 and IL-10 were maintained at the same levels and no significant difference was observed among all the groups. It is obvious that the level of IFN-γ were significantly higher than those of IL-4 and IL-10 especially in DNA/peptide regimen, which suggested the findings are typical of Th1-polarized signal, since the presence of IL-4 is a potent stimulus for Th2 differentiation. Therefore, these findings indicated specific Th1-type immune response was activated in response to TgGRA4, which are consistent with previous studies [17,28].

The main purpose of vaccination is to generate protective immune response against the infection of *T. gondii* and prolong the survival time of mice. In this study, 1 × 10^3^ RH tachyzoites was used to challenge immunized BALB/c mice. The RH strain of *T. gondii* is highly virulent in mice and no previous vaccines have been reported to provide entirely protect against intraperitoneal challenge with *T. gondii*[38,57]. In contrast to the non-immunized mice, a significantly prolonged survival time was obtained in all immunized mice. In addition, the survival rate of the DNA/peptide group reached to 40%, whereas mice in other groups died within 20 days. In all vaccination strategies, DNA/peptide produces an effective and significant degree of protection, and the effective and strong immunity acquired in the vaccination is capable of protecting the mice from *T. gondii* lethal acute challenge infection. Although the immunizations did not provide complete protection against acute challenge with *T. gondii*, the survival time of mice is longer and the survival rate was significantly increased than control groups. In this study, we mainly analyze a Th1-biased response to evaluate the efficiency of vaccine, which is the major limitation of our current study design. The immune response elicited by vaccine in mice was complex, and it is not sufficient to merely analyze a Th1-biased response. In the future study, evaluating the efficiency of vaccine from different aspects could be a new and promising strategy. In addition, many factors, such as *T. gondii* strain, the dose of inoculum, the inoculation route, and the mouse strain, might hinder an efficient evaluation of *T. gondii* vaccines [58]. It is known that intraperitoneal infection with tachyzoites is an unnatural route though it can easily be performed. Thus, in some immunization studies, oral challenge, the natural route of infection, were made with cystogenic strains [59]. Therefore, in our study, many parameters might hinder the effective evaluation of protective immunity. The current study is the lack of a full and systematic evaluation of the immune response generated by different experimental parameters. In further studies, considering different aspects and optimizing challenge route could achieve more encouraging results and effectively evaluate the immune response elicited by *T. gondii* vaccine.

## Conclusions

In the present study, we have demonstrated that TgGRA4 can induce significant humoral and cellular Th1 immune responses using a heterologous prime-boost vaccination strategy. The results suggested DNA prime-peptide boost vaccination elicited stronger humoral and cellular immune responses against *T. gondii* as well as increased the survival rate significantly compared with the controls. Therefore, this DNA prime-peptide boost immunization regime based on GRA4 may be an important approach to prevent *T. gondii* infection, particularly with respect to generating an efficient protective immune response.

## Competing interests

The authors declare that they have no competing interests.

## Authors’ contributions

SH and AZ conceived and designed the study, and contributed to the revision of the manuscript. MM carried out the experiments and drafted the manuscript. GL and LW participated in mouse immunization and challenge. GZ, YH, HZ, HC, QZ and XQZ helped in study implementation and data collection. All authors read and approved the final manuscript.

## Pre-publication history

The pre-publication history for this paper can be accessed here:

http://www.biomedcentral.com/1471-2334/13/494/prepub
